# Tumor-related gene expression levels in pulmonary pleomorphic carcinoma

**DOI:** 10.1186/s13019-015-0282-1

**Published:** 2015-06-03

**Authors:** Takeshi Oyaizu, Yuji Matsumura, Satoru Kobayashi, Tetsu Sado, Hiromi Ishihama, Masayuki Chida

**Affiliations:** 1 Department of General Thoracic Surgery, Dokkyo Medical University, Mibu, 321-0293 Japan; 2 Department of Cardiovascular and Thoracic Surgery, Dokkyo Koshigaya Hospital, 2-1-50 Minamikoshigaya, Koshigaya, Saitama 343-8555 Japan

**Keywords:** Lung cancer, Pleomorphic carcinoma, Gene expression

## Abstract

**Background:**

Pulmonary pleomorphic carcinoma (PPC) is a rare type of non-small-cell lung cancer that belongs to the family of sarcomatoid carcinomas and is associated with poor prognosis. We investigated the expressions of tumor-related genes in resected PPC specimens.

**Methods:**

Specimens resected from patients with PPC from July 2006 through April 2012 were investigated. Tumor segments were collected from the specimens by micro-dissection to extract mRNA, then RT-PCR was performed according to Dannenberg’s tumor profile method for semi-quantitation of tumor-related gene mRNA. To compare with other types of lung cancer, data from stage-matched adenocarcinoma (AC) and squamous cell carcinoma (SCC) cases in our database were also examined.

**Results:**

The gene expression levels of thymidylate synthase were significantly higher in PPC and SCC as compared to the AC specimens (*p* < 0.001). The levels of dihydropyrimidine dehydrogenase and thymidine phosphorylase mRNA in PPC showed a similar tendency to those in SCC, in contrast to AC. Furthermore, the expression level of excision repair cross-complementation group 1 mRNA in PPC specimens was similar to that reported in NSCLC, while the level of vascular endothelial growth factor (VEGF) expression was higher as compared to that reported for colorectal cancer.

**Conclusions:**

Although gene expression of tumor cannot be directly correlated to its sensitivity for anti-cancer drugs, it is likely that PPC tumors are not sensitive to anti-metabolic drugs. Anti-VEGF therapy may be a candidate for PPC, while cisplatin also remains a viable option.

## Background

A pulmonary pleomorphic carcinoma (PPC) is a rare type of non-small-cell lung cancer (NSCLC) that belongs to the family of sarcomatoid carcinomas [1] and is associated with poor prognosis [2–4]. Most affected patients experience recurrence and die during the first year after surgical intervention [2]. In general, the chemo-sensitivity of PPC is considered to be lower than that of other types of NSCLC, though Kawano et al. conducted a histoculture drug response assay and reported that some anticancer drugs may be effective [3]. Recently, the expression of various tumor-related genes have been reported to correlate to chemo-sensitivity and prognosis in several types of cancer [5–7]. However, few reports have been published regarding tumor-related gene expression levels in PPC. In the present study, we investigated the expressions of tumor-related genes in this rare tumor to establish an appropriate treatment strategy.

## Methods

Resected specimens from patients with PPC who underwent lung resection from July 2006 through April 2012 at our institutions were investigated. Those from patients who received preoperative induction therapy including chemotherapy or chemoradiotherapy were excluded. To compare with other types of lung cancer, 40 stage-matched adenocarcinoma (AC) and 35 squamous cell carcinoma (SCC) cases in our database [8] were also examined. Dokkyo Medical University Hospital Ethics Committee approved this retrospective study (#24035) and waived the need for patient consent for analysis of the results.

### Determination of tumor-related gene expression

Excised specimens were thinly sliced, then tumor segments were collected by micro-dissection to extract mRNA. RT-PCR was performed according to Danenberg’s tumor profile (DTP) method [9] for semi-quantitation of mRNA of tumor-related genes (Response Genetics Inc., New York, USA), including thymidylate synthase (TS), dihydropyrimidine dehydrogenase (DPD), thymidine phosphorylase (TP), excision repair cross-complementation group 1 (ERCC1), vascular endothelial growth factor (VEGF), and topoisomerase-1 (TOPO1). In brief, a laser captured micro-dissection technique was used on 10-μm sections with a magnification of 100x to obtain only cancer cells. Next, 1500–2000 round spots 80 μm in diameter were dissected for each case, from which total RNA was extracted. Real-time quantitative reverse transcription-PCR assays were performed on those samples using an ABI 7700 and TaqMan Probes. The level of ß-actin mRNA was used as a reference gene. Relative gene expression values are expressed as a ratio of PCR products of the gene of interest to that of the internal reference gene ß-actin.

### Statistics

Values are shown as the mean ± SD. Analysis of variance (ANOVA) was used for comparing among 3 groups, then Tukey’s post-hoc test was used when significance was found. Differences were considered significant at *P* < 0.05, with borderline significance considered at *P* < 0.10.

## Results

Among 536 patients who underwent surgery for primary lung cancer at our institution, 10 had PPC (1.9 %). They consisted of 9 men and 1 woman, with a mean age of 64.9 years (range 47–80 years). Patient characteristics are shown in Table 1.

**Table 1:** Patient characteristics

Case	Age/Sex	Stage	Chemotherapy	Relapse		Status
1	70/M	T2bN1M0	(+)	Distant	16 mo	Dead
			AMR			
2	75/M	T2bN1M0	(−)	(−)	39 mo	Alive
3	57/M	T2aN0M0	(+)	Distant	42 mo	Alive
			AMR, CBDCA + PTX			
4	77/M	T2bN0M0	(−)	Distant	9 mo	Dead
5	68/M	T2aN0M0	(+)	(−)	57 mo	Alive
			DTX + TS1			
6	80/M	T3N0M0	(−)	Distant	57 mo	Alive
7	54/M	T3N0M0	(−)	Local^a^	2 mo	Dead
8	47/M	T2bN0M0	(+)	Distant	12 mo	Dead
			TS1, CDDP + VNR, CBDCA + PTX			
9	48/F	T2aN1M1	(+)	Distant	14 mo	Dead
			CBDCA + DTX			
10	73/M	T2bN0M0	(−)	(−)^b^	2 mo	Dead

Each stage is presented based on the 7th UICC TNM classification

*mo* months, *AMR* amrubicin, *CBDCA* carboplatin, *PTX* paclitaxel, *TS1* tegafur gimeracil oteracil, *DTX* docetaxel, *VNR* vinorelbine

^a^incomplete resection

^b^operative death due to cerebral infarction

**Fig. 1:**
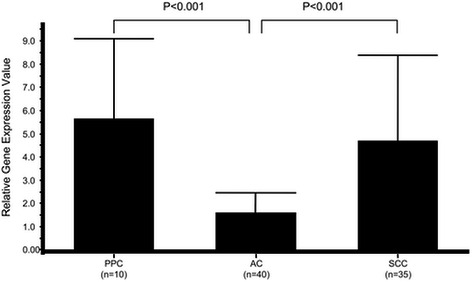
Comparison of relative gene expression of thymidylate synthase in cancer specimens. PPC, pulmonary pleomorphic carcinoma; AC, adenocarcinoma; SCC, squamous cell carcinoma

**Fig. 2:**
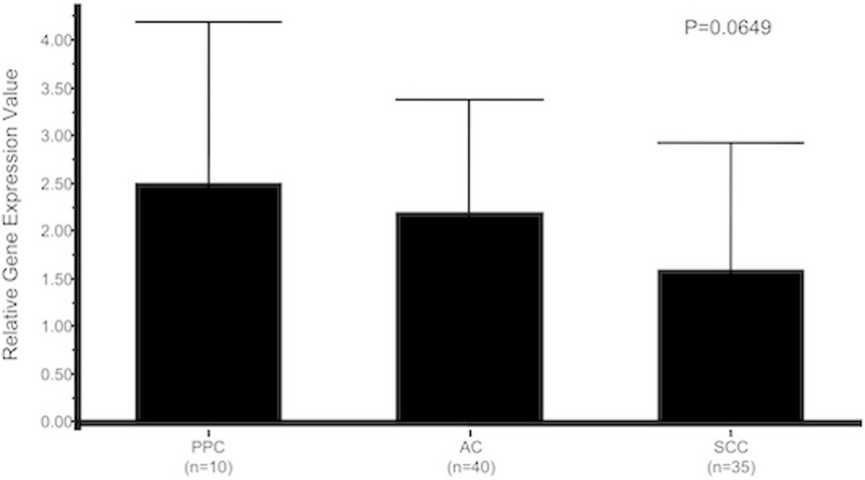
Comparison of relative gene expression of dihydropyrimidine dehydrogenase in cancer specimens. PPC, pulmonary pleomorphic carcinoma; AC, adenocarcinoma; SCC, squamous cell carcinoma

**Fig. 3:**
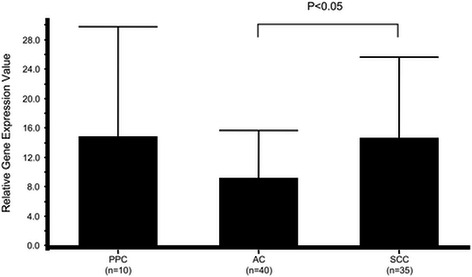
Comparison of relative gene expression of thymidine phosphorylase in cancer specimens. PPC, pulmonary pleomorphic carcinoma; AC, adenocarcinoma; SCC, squamous cell carcinoma

Gene expression levels of the nucleotide-metabolism-related enzymes TS, DPD, and TP in PPC were compared to those in AC and SCC specimens. The relative gene expression value of TS was 5.65 ± 3.46 in PPC, 1.59 ± 0.86 in AC, and 4.68 ± 3.71 in SCC (Fig. 1). That value was significantly higher in PPC and SCC specimens as compared to AC (*p* < 0.001). In contrast, the relative gene expression value for DPD was 2.49 ± 1.70, 2.20 ± 1.18, and 1.59 ± 1.34, respectively (Fig. 2), which was greater in PPC and AC than in SCC, with borderline significance (*p* = 0.0649). That of TP in the specimens was 14.81 ± 14.99, 9.17 ± 6.51, and 14.61 ± 11.06, respectively (Fig. 3), with no significant difference between AC and SCC, while that of AC was significantly lower as compared to SCC (*p* < 0.05).

Other tumor-related gene expression levels are shown in Table 2. The relative gene expression values of ERCC1, VEGF, and TOPO1 in the PPC specimens were 1.68 ± 0.66, 8.53 ± 6.44, and 2.55 ± 0.73, respectively.

**Table 2:** Mean gene expression values of ERCC1, VEGF, and TOPO1 in PPC and other organ carcinoma specimens by DTP method

		ERCC1	VEGF	TOPO1
NSCLC [6]	*n* = 283	1.65	-	-
Colorectal cancer [7]	*n* = 31	-	3.79	-
Thymic cancer [12]	*n* = 14	3.62	10.06	4.02
PPC (present study)	*n* = 10	1.68	8.53	2.55

*ERCC1* excision repair cross-complementation group 1, *VEGF* vascular endothelial growth factor, *TOPO1* topoisomerase-1, *PPC* pulmonary pleomorphic carcinoma, *DTP* Danenberg’s tumor profile

## Discussion

PPC is a rare type of tumor and accounted for only 1.9 % of resected lung cancer cases examined in the present study. Although affected patients are known to have poor prognosis, scant research has been conducted on this tumor. This is the first known investigation of tumor-related gene expression in resected PPC specimens. Our results showed a significant increase in the level of TS mRNA in PPC compared to AC specimens.

The gene expression levels of the nucleotide-metabolism-related enzymes TS, DPD, and TP in PPC were compared to those in stage-matched NSCLC cases listed in our database. Anti-metabolic drugs, such as pemetrexed, gemcitabine, and 5-fluorouracil and its derivatives, are widely used as cancer chemotherapy agents, and their effects include inhibition of TS, as well as incorporation of its metabolites into RNA and DNA. Lower TS activity is thought to be correlated with greater sensitivity to anti-metabolic drugs [8, 9]. The present findings showed that gene expression levels of TS mRNA in PPC were significantly higher than in AC, whereas those of DPD and TP mRNA in PPC were similar to those in SCC, in contrast to AC. Generally, AC is thought to be more sensitive to anti-metabolic drugs as compared to SCC. Our results indicate that it is unlikely that PPC is sensitive to anti-metabolic drugs, which agrees with clinical experience.

The expression levels of other tumor-related genes were also examined. Unfortunately, we were not able to examine stage-matched AC and SCC specimens, thus used previously reported data (Table 2).

ERCC1 protein is involved in nucleotide excision repair of damaged DNA and determination of ERCC1 mRNA expression may have be clinically useful for cancer treatment, because one of the mechanisms of resistance to platinum chemotherapy drugs is correlated with high ERCC1 activity [10, 11]. We found that the gene expression level of ERCC1 mRNA in PPC specimens was similar to that reported in NSCLC. However, findings are limited and do not fully reveal the efficacy of platinum-based chemotherapy, of which cisplatin is a likely candidate for PPC treatment.

VEGF is a signal protein that stimulates angiogenesis and anti-VEGF therapies are important for treatment of certain cancers, because solid tumors do not grow beyond a limited size without an adequate blood supply provide by angiogenesis using VEGF signals. VEGF gene expression has been shown to be higher than that of colorectal cancer, thus anti-VEGF therapy may useful for PPC patients. Furthermore, TOPO-1, involved in cell division, is a target of some anti-cancer drugs, such as irinotecan, topotecan, and camptothecin. Nevertheless, there are few reports of a correlation between clinical outcome and TOPO-1 mRNA level determined by the DTP method, though TOPO-1 gene expression was found to be lower than that of thymic cancer.

The present study is limited by its design as an institutional report of a small population, due to the rarity of the disease. Additional studies and case accumulation are necessary.

## Conclusion

Gene expression levels of nucleotide-metabolism-related enzymes in PPC showed a pattern similar to those in SCC, while they were different as compared to AC specimens. Although gene expression of tumor cannot be directly correlated to its sensitivity for anti-cancer drugs, it is unlikely that PPC is sensitive to anti-metabolic drugs. On the other hand, anti-VEGF therapy may be effective for PPC, while cisplatin also remains a viable candidate.
